# Comparative Phylogeography Highlights the Double-Edged Sword of Climate Change Faced by Arctic- and Alpine-Adapted Mammals

**DOI:** 10.1371/journal.pone.0118396

**Published:** 2015-03-03

**Authors:** Hayley C. Lanier, Aren M. Gunderson, Marcelo Weksler, Vadim B. Fedorov, Link E. Olson

**Affiliations:** 1 University of Alaska Museum, University of Alaska Fairbanks, Fairbanks, Alaska 99775, United States of America; 2 Institute of Arctic Biology, University of Alaska Fairbanks, Fairbanks, Alaska 99775, United States of America

## Abstract

Recent studies suggest that alpine and arctic organisms may have distinctly different phylogeographic histories from temperate or tropical taxa, with recent range contraction into interglacial refugia as opposed to post-glacial expansion out of refugia. We use a combination of phylogeographic inference, demographic reconstructions, and hierarchical Approximate Bayesian Computation to test for phylodemographic concordance among five species of alpine-adapted small mammals in eastern Beringia. These species (Collared Pikas, Hoary Marmots, Brown Lemmings, Arctic Ground Squirrels, and Singing Voles) vary in specificity to alpine and boreal-tundra habitat but share commonalities (e.g., cold tolerance and nunatak survival) that might result in concordant responses to Pleistocene glaciations. All five species contain a similar phylogeographic disjunction separating eastern and Beringian lineages, which we show to be the result of simultaneous divergence. Genetic diversity is similar within each haplogroup for each species, and there is no support for a post-Pleistocene population expansion in eastern lineages relative to those from Beringia. Bayesian skyline plots for four of the five species do not support Pleistocene population contraction. Brown Lemmings show evidence of late Quaternary demographic expansion without subsequent population decline. The Wrangell-St. Elias region of eastern Alaska appears to be an important zone of recent secondary contact for nearctic alpine mammals. Despite differences in natural history and ecology, similar phylogeographic histories are supported for all species, suggesting that these, and likely other, alpine- and arctic-adapted taxa are already experiencing population and/or range declines that are likely to synergistically accelerate in the face of rapid climate change. Climate change may therefore be acting as a double-edged sword that erodes genetic diversity within populations but promotes divergence and the generation of biodiversity.

## Introduction

Pleistocene glacial cycles have dramatically shaped the spatial distribution of genetic variation in many species [1]. These massive shifts in climate drove repeated cycles of population contraction and expansion, affecting not only the amount of intraspecific genetic diversity but also its spatial distribution (e.g., [2,3]). While observations of the intraspecific effects of past climate cycling are common, species responses to that cycling are by no means uniform in space, time, or scope. Species responses to climate shifts are affected by many factors, including habitat availability, life history, ecology, and demography. As a result, some extant communities may have shifted together in response to glacial cycling, thus sharing similar patterns of intraspecific diversity across species, or they may represent recent assemblages with no Pleistocene analogs [4], in which case intraspecific diversity will be uncorrelated between species. Understanding how and where diversity has been partitioned is fundamental to modeling responses to past climate change and predicting the effects of future change [5]. This is particularly important for alpine- and arctic-adapted species, which may be at disproportinate risk due to warming temperatures [6].

**Fig 1:**
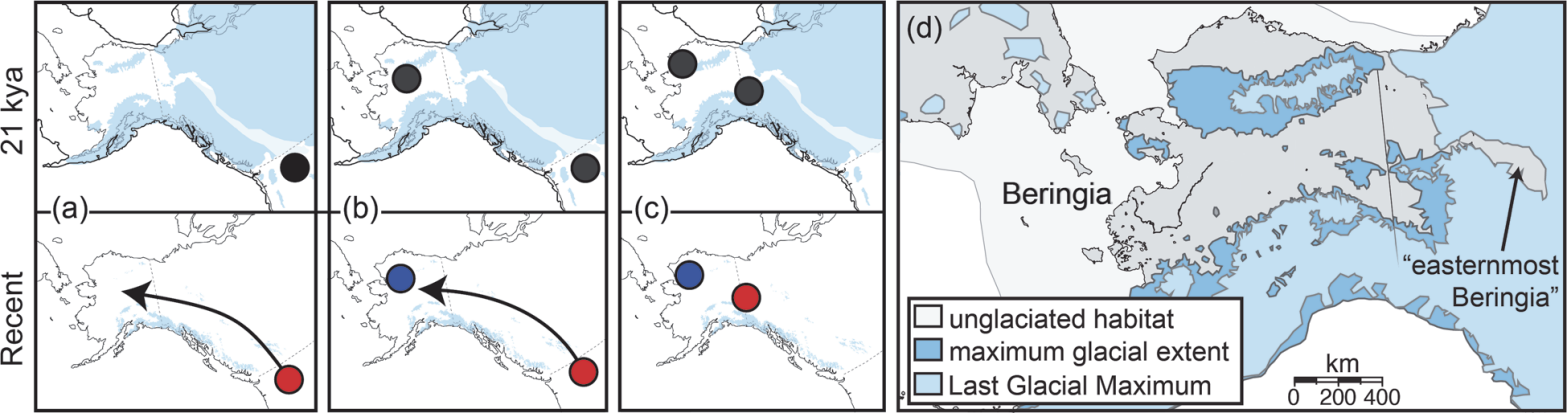
Beringian phylogeographic patterns. Patterns of ancestral areas and colonization routes (arrows) inferred for alpine mammals of eastern Beringia, modeled after [21]. A–C) Panels above show the ice sheet extent (blue) at the last glacial maximum, the ice-free corridor (light blue) thought to have opened between the Laurentide and Cordilleran ice sheets around 14 kya [22], relative Pleistocene population locations under each scenario. Suggested diversification and post-glacial colonization routes (arrows) shown below under a history of: a) sub-Laurentide colonization, b) vicariance between Beringian and sub-Laurentide populations, and c) intra-Beringian diversification. d) Available Beringian habitat during the Pleistocene. d) Glacial margins and ice-free areas of the Bering Land Bridge and Easternmost Beringia [23,24].

During previous glacial intervals the Laurentide and Cordilleran ice sheets blanketed much of North America, isolating plant and animal species in multiple refugia (Fig. 1). North of the ice sheets was Beringia, the vast ice-free corridor connecting North America and Asia [7,8]. Beringia has strongly influenced both Asian and North American diversity by permitting biotic interchange [9], limiting dispersal [10], and serving as a center of endemism in its own right [11–13]. Although Pleistocene speciation models have been rejected for a number of North American taxa (e.g., [14]), including some high-latitude alpine mammals [15], glacial separation and subsequent reunification of populations has resulted in introgression, competition, and/or replacement between divergent lineages within species [16–19]. Furthermore, cycles of repeated isolation, localized extirpation, and ensuing recolonization depressed genetic diversity in many northern species relative to their southern relatives [16,20].

In addition to the Beringian refugium, several cryptic northern refugia appear to have played an important role in the recolonization of previously glaciated regions for some cold-tolerant species [17,25]. These areas include “easternmost Beringia” (Fig. 1D; [23]), nunataks (emergent rock “islands” surrounded by glaciers and supporting plant and animal life; [26]) found throughout the region, and areas between adjacent ice sheets in northern British Columbia [27] that may have served as refugia for alpine species. Dominated by herbs and shrubs during previous glaciations [24], these regions appear to have maintained Pleistocene populations of alpine plants [28].

### Beringian phylogeographic patterns

Phylogeographic studies suggest that the majority of the recently colonizing species in eastern Beringia (present-day Alaska) arrived via North America [18,21]. Patterns of gene flow are related to ecology, with cold-adapted species exhibiting more genetic variation and more evidence of Beringian origins, whereas boreal and temperate forest taxa appear to be recent colonizers [17,21,25]. To date, three major phylogeographic patterns have been proposed for alpine mammals of eastern Beringia (Fig. 1; adapted from [21]): a) recent colonization from sub-Laurentide populations (i.e., those occurring south of the Laurentide and Cordilleran Ice Sheets shown in top panels of Fig. 1, B)) vicariant divergence between Beringian and sub-Laurentide populations followed by a northward expansion of southern isolates, and c) divergence between populations within Beringia.

**Fig 2:**
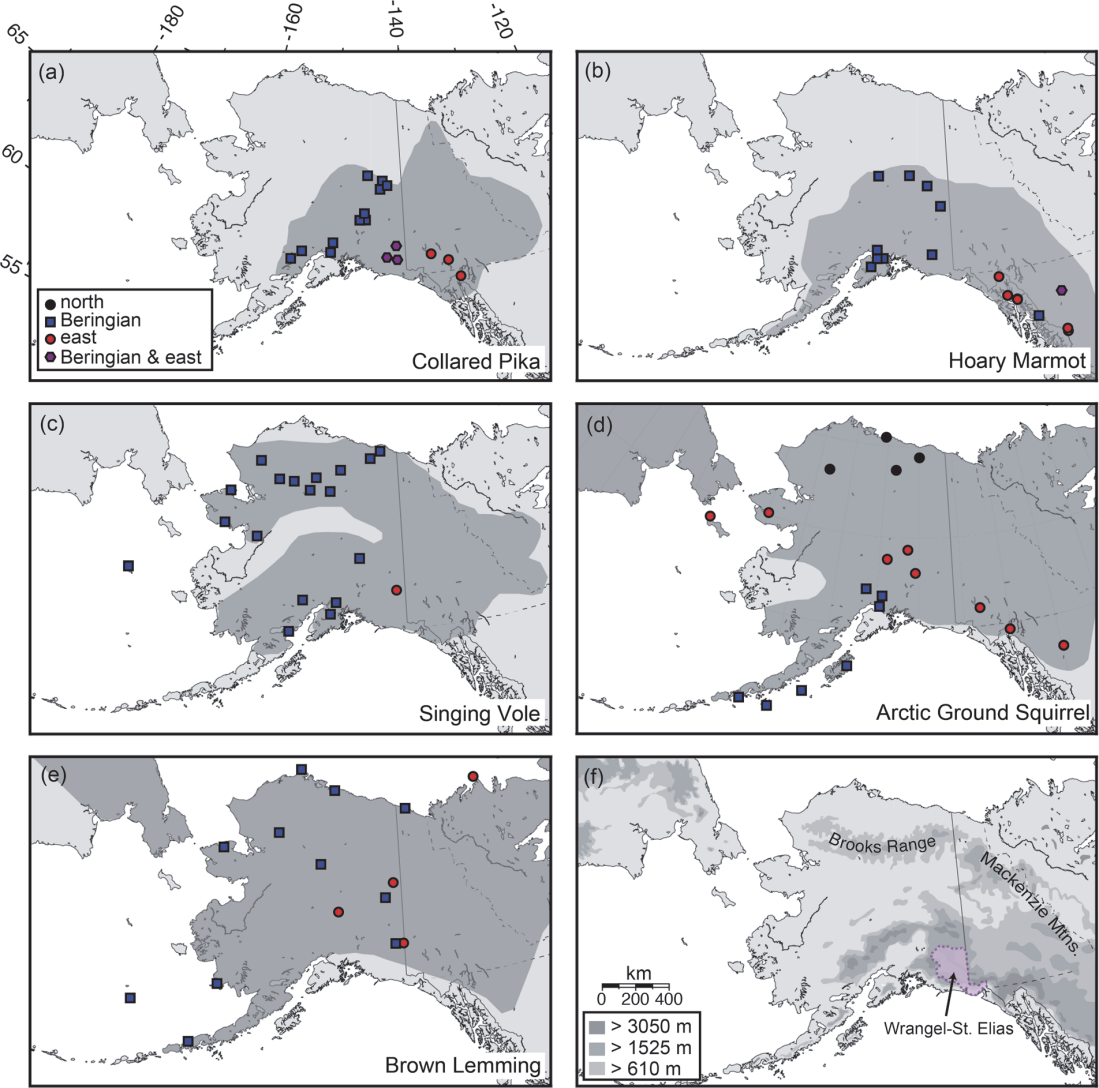
Species range maps, sampling, and lineage distributions. A–E) Range maps (dark grey) and sampling localities showing eastern (circles), Beringian (squares), and northern (black circles, Arctic Ground Squirrels only) groups, and populations with both east and Beringian haplotypes (hexagons). Range maps were modified from [35]. F) High elevation regions make up much of our study region, but the Brooks Range (in Northern Alaska) is also an extensive region of higher elevation habitat.

For several species that underwent vicariance between Beringian and sub-Laurentide or high-arctic populations, evidence of secondary contact between phylogroups has been detected [29–31]. Fedorov *et al*. [32] found that Nearctic Brown Lemmings (*Lemmus trimucronatus* Richardson, 1825; [Brown Lemmings hereafter]) fell into two major phylogeographic groups: a well-supported Beringian clade (hereafter B) currently found in Alaska and northeastern Russia and a Canadian Arctic/eastern Alaska clade to the east (E). Taking into consideration fossil records, they concluded that the eastern lineage was previously isolated south of the Laurentide ice sheet (Fig. 1B) [32]. In contrast, studies of the co-occurring alpine and arctic-tundra specialist, the Arctic Ground Squirrel (*Urocitellus* [formerly *Spermophilus*] *parryii* [Richardson, 1825]), identified three distinct mtDNA lineages that may have diversified in Beringia [33,34], suggesting a long history of isolation and diversification within the region. (Fig. 1C; [21]). Phylogeographic patterns in the Singing Vole (*Microtus miurus* Osgood, 1901) [21] and Collared Pika (*Ochotona collaris* Nelson, 1893) [20] are more ambiguous, with evidence of several successive population fluctuations within Beringia as well as divergence of that lineage (in each species) from one occurring in the Wrangell-St. Elias Mountains (Fig. 2F). However, with only two Singing Voles from the Wrangell Mts. lineage, Weksler *et al*. [21] were unable to distinguish between intra-Beringian divergence (Fig. 1C) and a history of sub-Laurentide vicariance with subsequent recolonization (Fig. 1B).

### Alaskan alpine small mammals

Recent research suggests that the range contraction/post-glacial expansion model commonly applied to temperate species may not be applicable to cold- and alpine-adapted species [19,36,37]. Extensive glaciations in the north (Fig. 1D) may have greatly limited the amount and extent of suitable habitat; however, a surprising number of species are known to be able to persist on debris-covered glaciers and nunataks [38]. Although the five species (Table 1) in our study vary in their affiliation with alpine and boreal-tundra habitat (S1 Table), we might expect them to demonstrate concordant phylogeographic and demographic patterns shaped by common historical events (e.g., glaciation; Fig. 1D), including expansion during glacial cycles and contraction in interglacials. First, they occur and thrive above treeline [39,40], along glacial margins, and on nunataks [41,42]. Second, four of the species—Singing Voles, Brown Lemmings, Arctic Ground Squirrels, and Collared Pikas—are well known from Pleistocene fossil localities in Alaska [43], suggesting they may have been affected by similar events. While fossil marmots are known from Alaska, none are reliably identifiable to species and definitive evidence is ambiguous for Hoary Marmots (*Marmota caligata* Eschscholtz, 1829), which are difficult to distinguish morphologically from the Alaska Marmot (*M*. *broweri*; [44]). Admittedly, the presence of fossils from the region does not rule out the possibility of extirpation and subsequent recolonization (e.g., [45]), but it is indicative of the potential for similar responses to past climate change.

**Table 1:** Beringian history.

**Species**	**Sister species**	**Proposed phylogeographic history**	**Beringian fossils**[50, 51]	**Nunatak survival**
**Hoary Marmot**	North America	post-Laurentide colonizer [25]	Late Pleistocene	yes [52]
	*M*. *vancouverensis* [46]	Fig. 1A		
**Singing Vole**	North America	Beringian endemic and colonizer [21,25]	Early to Middle Pleistocene	unknown
	*M*. *xanthognathus* [47]	Fig. 1B		
**Brown Lemming**	Asia	Beringian endemic and colonizer [17,32]	Early to Middle Pleistocene	yes [30]
	*L*. *sibiricus* [32]	Fig. 1B		
**Arctic Ground Squirrel**	North America	Beringian endemic [17,33]	Early to Middle Pleistocene	yes [42]
	*S*. *richardsoni* or *S*. *elegans* [48]	Fig. 1C		
**Collared Pika**	North America	Beringian endemic [15,49]	Early to Middle Pleistocene	yes [42]
	*O*. *princeps*[15]	Fig. 1C		

Geographic location of sister species, phylogeographic histories (see Fig. 1) proposed by previous studies, presence of fossils from Beringia, and documented nunatak survival for the five species included in this study.

Despite similarities between species, concordant responses to past climate change are generally not the norm [53]. Contrasting the phylogeographic patterns of co-distributed species with differing ecological requirements allows the examination of similarities in both the geographic distribution of genetic variation and timing of population divergence. In terms of timing, species diversifying within Beringia (Fig. 1C) may have diverged simultaneously or nearly simultaneously with one another but asynchronously compared to species that experienced vicariance between Beringian and sub-Laurentide populations (Fig. 1B). Our dataset affords an opportunity to test for guild-wide responses to past change and to determine if alpine-adapted small mammals responded in a uniform fashion to post-Pleistocene environmental shifts. This in turn might enable subsequent predictions regarding ongoing and future responses to climate change.

### Objectives

We seek a better understanding of the roles of climate shifts and the regional environment in shaping intraspecific genetic diversity across five alpine small mammals (Table 1: Arctic Ground Squirrels, Collared Pikas, Hoary Marmots, Brown Lemmings, and Singing Voles) that co-occur in Alaska and northwest Canada. Although some of these species have been characterized separately, applying a model-based comparative phylogeographic approach allows us to test for the relative influence of shared historical events and concerted evolutionary change across species [54]. Specifically, we tested whether co-distributed alpine small mammals responded demographically to Pleistocene climate change and if genetic diversity patterns are indicative of a common history of intra-Beringian diversification (Fig. 1C), vicariance between Beringian and sub-Laurentide populations (Fig. 1B), or patterns that differ idiosyncratically between species (Fig. 1A,B,C; Table 2). We also applied a coalescent modeling approach to determine if these cold-adapted species show evidence of range contraction into interglacial refugia as opposed to post-glacial expansion out of refugia. This has broad implications for understanding how organisms at high latitudes have responded to past climate change and is critical for predicting responses to ongoing and future shifts in climate.

**Table 2:** Hypotheses.

	**Intra-Beringian diversification**	**Vicariance between Beringia and sub-Laurentide populations**	**Species-specific patterns**
**Expected diversity**	B ≅ E	B >> E	B ≅ E, B >> E
**Expected demographic expansion**	B ≅ E	B << E	B ≅ E, B << E
**Expected number of divergence times**	1	1	>1

Expectations of relative genetic diversity, signal of range expansion, and the number of separate divergence times suggested for Beringian (B) and eastern (E) lineages under each historical model (corresponding to patterns in Fig. 1C and Fig. 1B, and to a no common pattern model, respectively).

## Materials and Methods

We assembled a dataset of mitochondrial cytochrome *b* sequences for five co-distributed small mammal species (Table 1) from alpine areas of eastern Beringia. Complete cytochrome *b* sequences (1140 bp) from published studies on Collared Pikas [20], Singing Voles [21], Arctic Ground Squirrels [33], and Hoary Marmots [44], as well as 915 bp of cytochrome *b* for Brown Lemmings [32] were retrieved from GenBank. Additional cytochrome *b* sequences for Hoary Marmots and Collared Pikas were generated from tissues archived at the University of Alaska Museum as described previously [15,55]. We included 77 Collared Pikas (representing 17 unique localities), 26 Hoary Marmots (15 unique localities), 37 Singing Voles (19 unique localities), 28 Brown Lemmings (14 unique localities), and 48 Arctic Ground Squirrels (19 unique localities) in the comparative phylogeographic dataset. Geographic distributions, phylogeographic subdivisions, and sample sizes are given in Fig. 2 and Table 3. Most of the samples are associated with voucher specimens in the University of Alaska Museum’s mammal collection (S2 File).

**Table 3:** Population genetics diversity metrics.

**Species**	**Sub-clade**	***n***	***h***	***S***	**θ_π_**	***Fs***	***H***	**Tajima's *D***
**Collared Pika**	*Beringian*	58	27	47	0.0058	**−8.192**	3.6515	−1.231
	*East*	19	12	19	0.0042	−3.23	3.187	−0.678
**Hoary Marmot**	*Beringian*	14	4	3	0.0009	−0.327	0.2637	0.326
	*East*	12	5	5	0.0012	−1.079	0.9697	−0.583
**Singing Vole**	*Beringian*	35	30	87	0.0121	**−12.499**	**−20.981**	−1.448
	*East*	2	2	2	0.0018	-	-	-
**Brown Lemming**	*Beringian*	13	13	49	0.0122	**−5.429**	−6.987	**−1.555**
	*East*	14	14	42	0.0121	**−6.188**	−9.1429	−0.874
**Arctic Ground Squirrel**	*Beringian*	21	8	26	0.0052	1.492	−6.89	−0.702
	*East*	20	12	39	0.0068	−1.012	0.2421	−1.19
	*North*	7	3	5	0.0023	1.853	1	1.28

Population genetic summary statistics: *n* = number of samples, *h* = number of haplotypes, *S* = number of segregating sites, θ based on π and a finite sites model, Fu’s *Fs*, Fay and Wu’s *H*, and Tajima’s *D*. Note that there are no values given for Fs, H, or D for the eastern Singing Vole lineage, because of an insufficient sample size. Test statistics significant at the p < 0.05 level (tested with 10,000 coalescent simulations in DnaSP), are shown in bold text.

### Phylogenetic and population genetic inference

Initially, we used phylogenetic trees and sampling maps to compare phylogeographic distributions. We used jModelTest [56,57] to compare 88 possible nucleotide substitution models on a maximum likelihood- (ML) optimized tree. We used the Akaike Information Criterion corrected for small sample sizes (AICc) to select between models [58]. While the small sample size correction may not be necessary for this dataset, AICc converges with AIC as sample size increases [59]. Heuristic tree searches and bootstrapping under the ML criterion were conducted using GARLI v. 0.942 [60]. Maximum-likelihood trees and models of evolution are given in S1 File (Supporting Figures). We classified Beringian (B; following [32]) and east (E; east of central Beringia) lineages as those phylogeographic groups (hereafter phylogroups) separated by the longest internode in each unrooted ML tree. These lineages correspond to mostly Beringian and mostly eastern geographic distributions, respectively. The northern lineage (N; Brooks Range, from northern Alaska; Fig. 2D) in Arctic Ground Squirrels was treated separately following [34].

Population genetic summary statistics (Table 3) were computed in DnaSP v. 5 [61]. To compare genetic diversity within clades, we computed the number of haplotypes (*h*), the number of segregating sites (*S*), and the per-site effective population size parameter (*θ*) based on π (nucleotide diversity) under a finite sites model [62]. To compare within-species divergences, we calculated the net number of nucleotide substitutions between E and B phylogroups (*D*_*a*_; [63]). Several different summary statistics were used to test for evidence of demographic expansion, including Tajima’s *D*, Fay and Wu’s *H*, and Fu’s *Fs* (see below). A standard statistical test used to identify selection and/or demographic history, Tajima’s *D* [64] compares two estimates of *θ* to look for departures from neutrality. Tajima’s *D* is particularly sensitive to demographic expansion [65]. Positive values are considered evidence of a recent population bottleneck whereas negative values are indicative of population expansion. We also calculated a second statistic, Fay and Wu’s *H*, which may be more sensitive than other metrics for small sample sizes [66]. Fay and Wu’s *H* contrasts low- and intermediate-frequency alleles and is more sensitive to population decline and population subdivision than Tajima’s *D* [65]. We contrast these two methods based on *θ* estimators with Fu’s *Fs*, a method based on the haplotype frequency distribution that may be more sensitive than Tajima’s *D* to population expansion [67]. Significance for each of these tests was calculated at a 95% level using 10,000 coalescent simulations in DnaSP.

### Demographic histories

While summary statistic calculations can be useful, coalescent- and simulation-based approaches take full advantage of the genealogical relationships among alleles [68]. To compare and contrast demographic histories within and between the alpine small mammals of eastern Beringia, we used a series of Bayesian skyline plots (BSPs) implemented in BEAST 1.4.8 [69]. This approach employs a flexible coalescent prior, without a pre-specified demographic history, to examine population fluctuations over time, with an emphasis on the past 10 time dimensions. For each species, we used the model of evolution determined by jModelTest. Three separate analyses were conducted (for 10, 20, and 50 million generations, respectively) to check for MCMC convergence. Trees and parameters were sampled every 1000 generations, and the initial 10% of the output was removed as burn-in. We used Tracer v. 1.5 to verify effective sample size and construct BSP intervals. Estimates of the relative timing of demographic change and glacial cycles were made using lineage-specific substitution rates calculated in previous studies (substitutions site^−1^ Myr^−1^): *Lemmus* 11.7% [70], *Marmota* 3% [71], *Microtus* 4% [72], *Ochotona* 3.14% (averaged from [15,73]), and *Urocitellus* 1.52% [33].

### Tests of temporal concordance

The relative timing of within-species divergence (i.e., between phylogroups) was examined using a hierarchical Approximate Bayesian Computation (hABC) implemented in msBayes v. 20100519 [74]. This flexible, simulation-based approach uses a hierarchical model to estimate the probable number of divergence events (Ψ) among a set of ‘species pairs’ [74,75] while explicitly incorporating uncertainty in population sizes, mutation rates, and demographic histories. To determine whether species divergences were the result of a single event (i.e., temporally congruent; Ψ = 1) or occurred independently (Ψ>1) we preformed two similar analyses that differed in the treatment of Arctic Ground Squirrels. The first analysis included representatives from each of the phylogroups (E-B split) from all five species, with the northern lineage of Arctic Ground Squirrels excluded. As Arctic Ground Squirrels comprise three clades instead of two and the origin of the northern lineage is controversial (e.g., [34]), the second analysis contrasted the timing of the split of the eastern plus central Beringian squirrel clades (E+B) from the northern clade (N) against the timing of the E-B split in the other taxa. Because of the simulation approach implemented in msBayes, datasets with small sample sizes (e.g., 2–5 individuals per population) can produce robust results [75]. As predicted under coalescent theory [76], datasets larger than 20 individuals per species pair increased the computation time and variance without greatly influencing the results.

The hierarchical model implemented in msBayes estimates a set of three hyperparameters (Ψ, the number of divergence times; E(*τ*), the mean divergence time; and Ω, the ratio of variance in divergence time to the mean in divergence times, Var(*τ*)/E(*τ*)), which characterize the variability of within population specific sub-parameters. These sub-parameters (population divergence time—*τ*_i_, current and ancestral population sizes, post-divergence population size, and post-divergence population growth) are allowed to vary independently among the population pairs (i.e., phylogroups). For both analyses we set upper and lower bounds on the prior for *θ* (4e-08 < *θ* < 0.0048652; based upon observed π within subpopulations), an upper bound on the time of separation *τ* (*τ* < 2), a uniform prior (0, 5) on the number of *τ* classes with different values (Ψ), and the default prior on the ancestral *θ* multiplier (0.5). Because we defined ‘species pairs’ as phylogroups within a species, the migration parameter was excluded. The msBayes program samples from the posterior distribution through generating a series of coalescent simulations and compiling a vector of six summary statistics (*π*, *θ*_w_, *π*_*net*_, *π*_*b*_, *π*_*w*_, and Var(*π-θ*_w_) [75]) for each eastern or western population based upon simulated dataset. These six statistics include the average number of pairwise differences among sequences within a population pair (*π*_*i*_), the number of segregating sites within each population (*θ*_w_), Nei and Li’s net nucleotide divergence between each pair of populations (*π*_*net*_), and average pairwise differences between (*π*_*b*_) and within a population (*π*_*w*_). This summary statistic vector is then compared to the observed summary statistics using an accept/reject algorithm to construct the posterior distribution for the three hyperparameters of interest (Ψ, E(*τ*), and Ω). We conducted 1,000,000 coalescent simulations for each analysis and used a tolerance of 0.00055 (the proportion of the posterior distribution used in the analysis) to yield approximately 550 draws from which to construct posterior intervals (based on recommendations by [75]). Sensitivity to the prior distributions was examined by two additional analyses: one to examine a scenario wherein ancestral population size is smaller, with the bounds on the ancestral *θ* prior decreased to 0.25, and another analysis in which the timeframe of divergences was widened by increasing the upper limit on τ to 10.0.

## Results

### Geographical concordance

**Fig 3:**
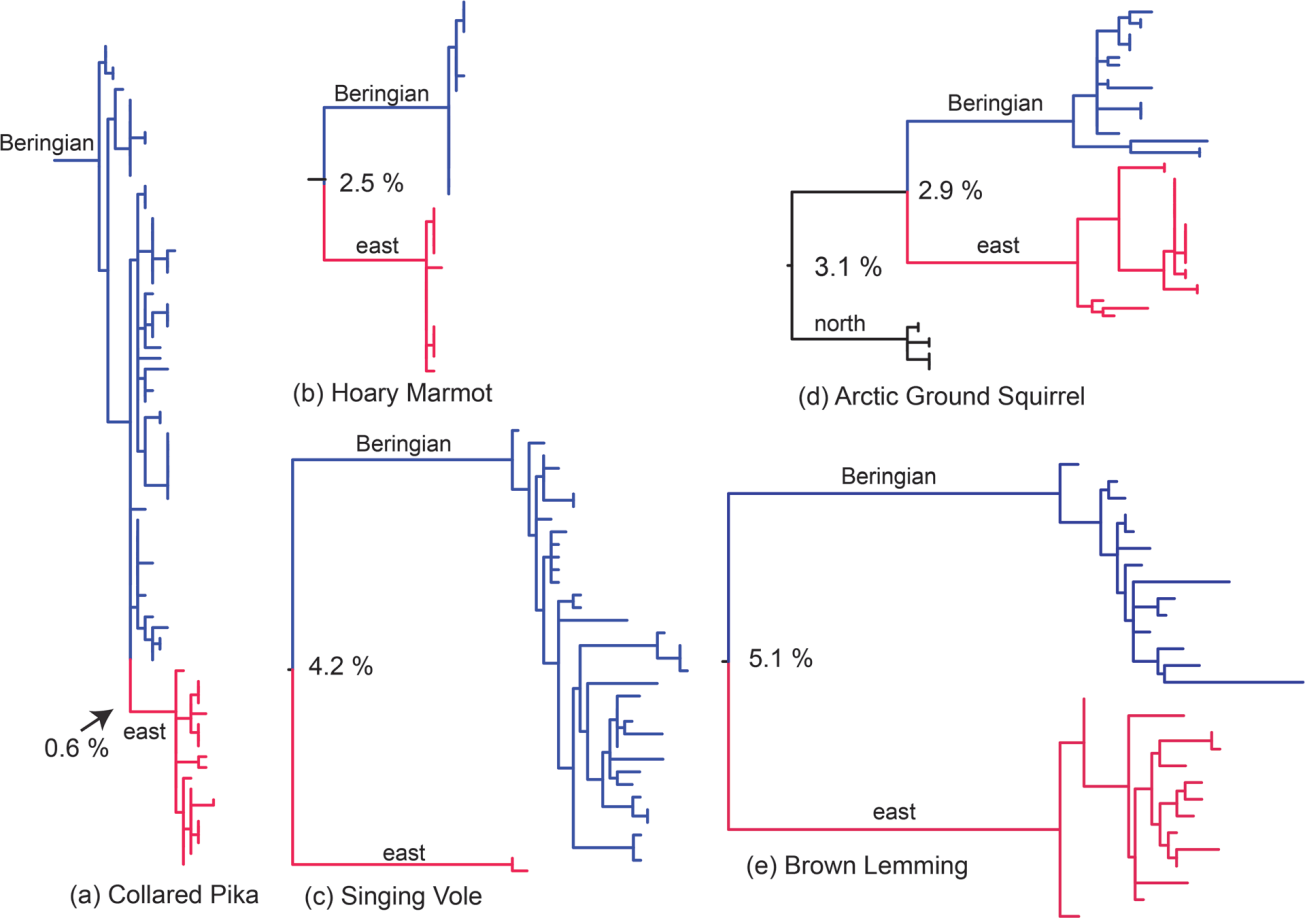
Scaled likelihood trees. Scaled likelihood trees for a) Collared Pikas, b) Hoary Marmots, c) Singing Voles, d) Arctic Ground Squirrels, and e) Brown Lemmings. Beringian (above, blue) and east (below, red) lineages are depicted for all species. The northern lineage of Arctic Ground Squirrels is sister to the east-Beringian phylogroups. Pairwise net divergences (*Da* calculated in DnaSP; [61]) are shown between phylogroups.

Phylogroups generally corresponding to Beringian (B) and eastern (E) lineages were recovered in all five species (Figs. 2 & 3). Relatively deep subdivision was apparent between phylogroups for Brown Lemmings and Singing Voles. Divergence between phylogroups was less (2.5%) for Hoary Marmots and much less (0.6%) for Collared Pikas (Fig. 3; S1 File). A relatively flat likelihood surface with respect to the placement of the root renders the Beringian populations of pikas polyphyletic with respect to an eastern clade. Arctic Ground Squirrels include two clades south of the Brooks Range, one with a more “eastern” distribution but with some longitudinal overlap on islands around Alaska (Fig. 2D), which appear to be due to recent introductions rather than historical range shifts [77]. As mentioned previously, there is a northern lineage of Arctic Ground Squirrels that is sister to the E-B lineage (Fig. 3D).

Geographically, the putative contact zones between clades appeared to vary widely (Fig. 2), although we consider our sampling too coarse to definitively identify contact or suture zones. Contact zones within Brown Lemmings, Collared Pikas, and Singing Voles are roughly coincident with Wrangell-St. Elias National Park and Preserve (NPP) and the Alaska-Canada border. The contact zone within Hoary Marmots was farther to the east, and both lineages are sympatric in northern British Columbia. Arctic Ground Squirrels contain regional phylogeographic patterns that appear dissimilar to the other four species (with a northern lineage and a broadly distributed eastern clade; Fig. 2), but this doesn’t rule out the possibility of temporal concordance with the other four species.

### Demographic inference and population fluctuation

The greatest degree of genetic divergence between phylogroups (Fig. 3) and genetic diversity within each (E or B) phylogroup was seen in Brown Lemmings and Singing Voles (Table 3). Values of *θ* within both Brown Lemming and the Beringian (B) Singing Vole phylogroups were approximately twice those in Collared Pikas and Arctic Ground Squirrels and ten times those in Hoary Marmots. The lowest values for the diversity metrics were observed in Hoary Marmots. None of the species showed evidence of eastern population expansion and Beringian demographic stability. On the contrary, only the Beringian group of Collared Pikas and Singing Voles and both lineages of Brown Lemmings showed significant expansion signals (based on significant values for Fu’s *Fs*). Tajima’s *D* (considered to be less sensitive than Fu’s Fs; [67]) indicated a significant population expansion in only Beringian Brown Lemmings. Fay and Wu’s *H* was significant only for the Beringian Singing Voles.

**Fig 4:**
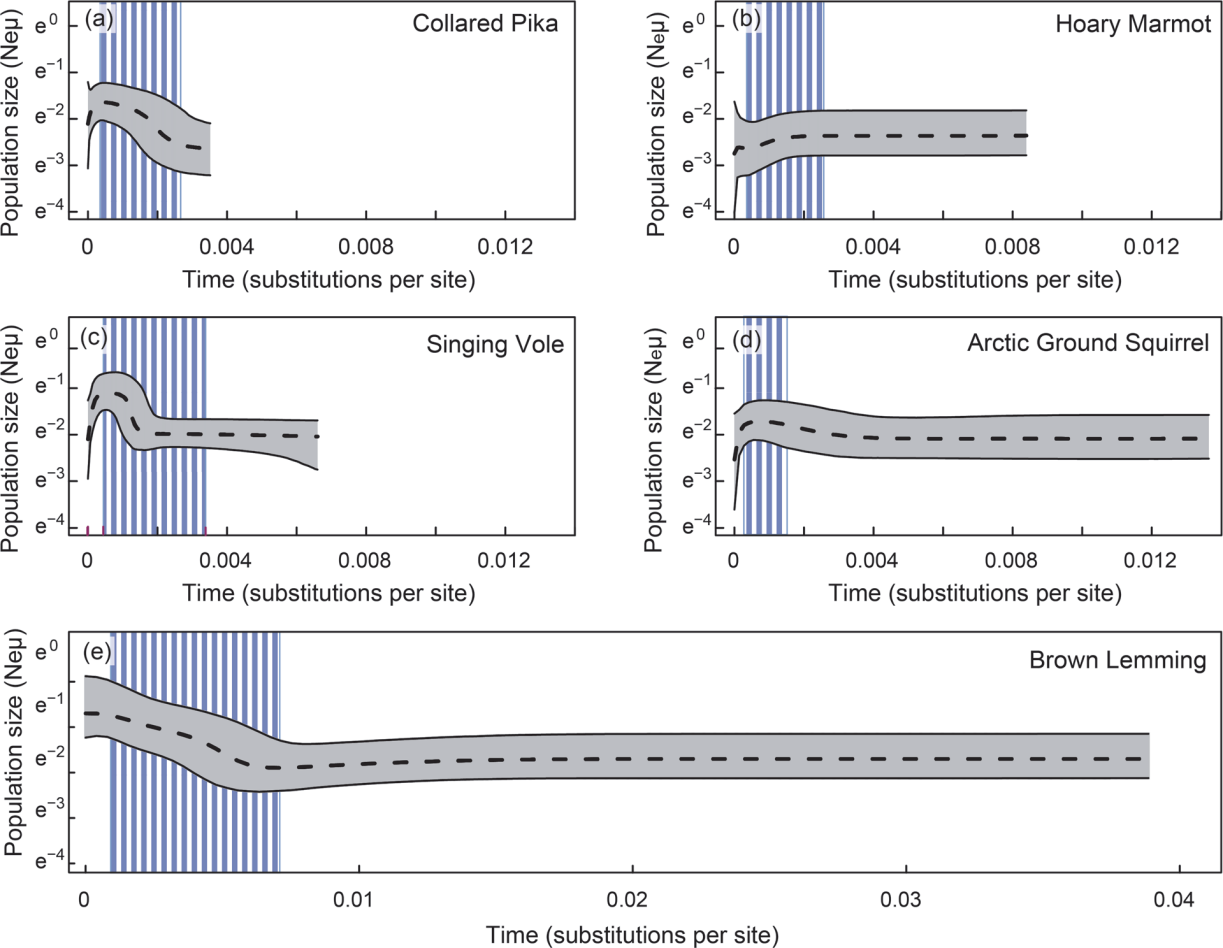
Past demographic shifts. Demographic change (in units of effective population size scaled by mutation rate) over time (in substitutions per site) for all five species shown using Bayesian skyline plots. Note the different x- and y- axes for Brown Lemmings (e), for which a longer population history and a larger scaled effective population size were suggested. The median population size is the center dashed line, the 95% highest posterior density (HPD) intervals are shown in surrounding grey, and the relative timing of the Wisconsin glaciation (based on taxon-specific mutation rates) is shown in blue. Because of the single locus and contemporaneous sampling utilized in this study, only recent demographic trends are evident.

Bayesian skyline plots (BSPs; Fig. 4) recovered a greater range of demographic signals than did summary statistics. Pleistocene range expansion followed by recent demographic contractions appears to have occurred in Singing Voles, Collared Pikas, and possibly Arctic Ground Squirrels. The BSP analysis indicated a strong signal of Pleistocene and recent demographic expansion as well as a higher (by 1–2 orders of magnitude) long-term effective population size for Brown Lemmings. There was no clear support for a trend in either direction in Hoary Marmots; however, the exceptionally low genetic diversity makes it difficult for coalescence-based methods to distinguish between alternate demographic trajectories with any confidence.

### Testing for simultaneous divergence

Despite differences in divergence depth between eastern and Beringian phylogroups (Fig. 3), hABC analyses generally supported simultaneous divergences for all five species, with a mode (Ψ_mode_) of 1.0 (Ψ_median_ = 1.6). The distribution of posterior probabilities was somewhat broader: 60.8% of the posterior probability supported simultaneous divergence within all five species, 23% supported two separate divergence times, and 12.2% supported three divergence intervals (all other divergences receiving <5% posterior support). If divergence was simultaneous, the ratio of variance to expected divergence time (Ω = Var[t]/E[t]) should approach zero. For this dataset the mode of the distribution was close to zero (Ω_mode_ = 0.04, 95% HPD interval: 0.0–0.32). Analyses with differing priors on tau or theta confirmed the robustness of this pattern, with the mode of the distribution for tau centered on 1. When the N-S_E+B_ split in Arctic Ground Squirrels was used instead of the E-W split, simultaneous divergence was even more strongly supported (Ψ_mode_ = 1; Ψ_median_ = 1.27; 79.6% of posterior probability supporting a single divergence, 14.8% supporting two divergences, and 4.2% supporting three divergences). The ratio of expected divergence times was similarly close to zero (Ω_mode_ = 0.02; 95% HPD interval: 0.00–0.17). While the modes of these distributions are similar, analyses including the N-E_E-B_ split in Arctic Ground Squirrels resulted a slightly longer tail on parameter distributions, encompassing greater variation in the range of Ψ, Ω, and the expected time to divergence.

## Discussion

### Biogeographic history of eastern Beringia

Our results support simultaneous intraspecific divergence between central Beringian (B) and eastern (E) populations. Hoary Marmots and Brown Lemmings contained approximately equal genetic diversity and equivalent expansion signals (or lack thereof in Hoary Marmots) between Beringian and eastern phylogroups (B ≅ E). These results refute a history of recent northern expansion in the eastern lineage (Fig. 1B) and instead indicate intra-Beringian diversification (Fig. 1C). In fact, none of the species showed evidence of population expansion in the eastern phylogroups without equal support for a Beringian phylogroup expansion. Although genetic diversity was reduced in the eastern lineages of Collared Pikas and Singing Voles relative to the Beringian lineages, this appears to be an effect of sample size rather than truly disparate diversity. Strong support for a temporally concordant divergence event suggests that all five species may have responded in a similar manner to climate shifts, with species-specific differences in local abundance and ancestral population size (Fig. 1A).

Pleistocene population centers within Beringia (Alaska) and northwestern Canada likely differed at fine scales by species and over time (accounting for the fact that multiple divergences received some posterior probability support in the hABC analyses), resulting in differential genetic diversity but broadly similar phylogeographic patterns (i.e., the presence of two geographically concordant lineages) across all five species). Spatial heterogeneity over time may explain differences in ancestral population size among phylogroups, such as the greater genetic diversity in eastern Arctic Ground Squirrels (E>B), which was not predicted under any of the models (Fig. 1A, B, or C). Population subdivision is also indicated in the contrast between Fay and Wu’s *H* and Tajima’s *D* in Singing Voles. This isolation in multiple microrefugia—small favorable regions of unglaciated habitat such as easternmost Beringia [24] or nunataks [78]—would have a strong effect on the phylogeographic patterns in the alpine mammals of eastern Beringia. Smaller and patchier populations along the ice sheet margins in easternmost Beringia during the Last Glacial Maximum (LGM) have recently been suggested by species distribution modeling for both pikas [20] and Arctic Ground Squirrels [34]. Suitable habitat during the LGM is also predicted for mountain goats in this region [79].

The hypothesis of simultaneous isolation of Beringian and sub-Laurentide populations and subsequent northward expansion of a small colonizing lineage (Fig. 1B) received little to no support in our analysis, as there is no sign of genetic reduction in the Eastern phylogroups, which would be expected under a recent colonization scenario [21]. Nevertheless, a demographic scenario of vicariance and subsequent northward expansion cannot be entirely excluded, particularly if such isolation and expansion predated the LGM. More recent northern migration with a high degree of population substructure and local (as opposed to long-distance) migration could also have maintained genetic diversity, thus masking the signal of northward population expansion. Simultaneous distributional shifts and founder events reducing effective population size have been hypothesized for rodent populations in Siberia [29,80]. Given the wide range of scenarios compatible with the data (e.g., support for simultaneous divergence with either Arctic Ground Squirrel split), this is certainly possible, particularly for Hoary Marmots, the only species with current populations found south of the former ice-sheet margins. However, a simultaneous, recent northward migration of all eastern clades for all species in our study seems unlikely based on the differences in natural history, dispersal ability, and local population structure.

### How simultaneous were divergences?

**Fig 5:**
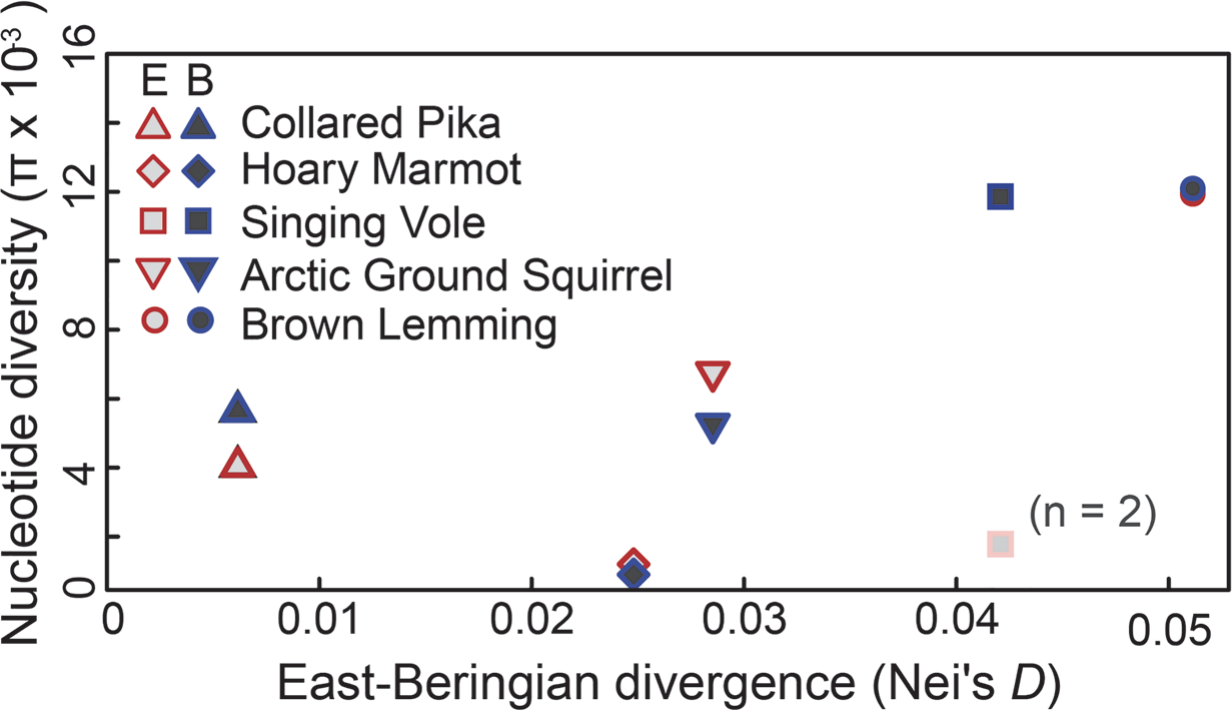
Divergence between clades within a species. Divergence (measured as Nei’s *D*) is generally correlated to within-clade diversity (*π*) for alpine mammals in this study. Hoary Marmots are the exception to this trend, demonstrating low within-clade diversity for the amount of between-clade divergence. Diversity levels in the eastern clade of Singing Voles are shown for reference but should be interpreted with caution as the clade is represented by only two samples.

Given the superficial differences in number of northern lineages and the percent divergence between phylogroups (0.6% in Collared Pikas to 5.1% in Brown Lemmings), the fact that a single simultaneous divergence in all five species was strongly supported may seem surprising. However, given sufficient differences in population size and mutation rate between species (i.e., differences in *θ*), the degree of genetic divergence between lineages separated by a single event can be highly variable (see [54]). In fact, when within-phylogroup divergence is contrasted with between-phylogroup divergence for each species, the accumulation of between-phylogroup divergence generally predicted within-phylogroup diversity in every species except Hoary Marmots (Fig. 5). Previous estimates (based on a molecular clock) for the timing of divergence within Singing Voles suggest that eastern and Beringian lineages may have shared a most recent common ancestor between 254–651 kya, a fairly wide swath of the Pleistocene relative to glacial cycles [21]. This overlaps with the wide interval suggested for Arctic Ground Squirrels (5 kya—1.157 mya; [34]).

It may seem counterintuitive that hABC analyses returned similarly strong support for simultaneous divergence whether the E-B split or the N-S_E+B_ split for Arctic Ground Squirrels was used. This may reflect simultaneous divergence between all three groups, or it may instead indicate that many coalescent histories can fit the data. A recent multi-locus study of Arctic Ground Squirrels reached a similar conclusion that several hypotheses provide a partial fit to the observed data [34]. Given the low levels of genetic variability in many loci (e.g., [20]), arctic species in particular may require genomic-level data to distinguish among specific phylogeographic histories. Time to divergence between lineages often decreases at higher latitudes (especially when considered relative to effective population size), and as a result, divergences within high-latitude lineages are generally smaller than their lower-latitude counterparts (e.g., [3,20]). If divergences between all three Arctic Ground Squirrel clades (E, B, and N) were simultaneous no temporal difference would be detected in our analysis. Within- and between-group diversity metrics for Arctic Ground Squirrels do not differ from those of the other species under a model of simultaneous divergence (Fig. 5). Given that hABC techniques gain additional power from comparing across multiple species pairs, they are able to distinguish between non-simultaneous divergent events among other recently-diverged northern species/population pairs (e.g., [81,82]); this continued support for a single simulataneous divergence in our data is therefore unlikely to be a methodological artefact.

### Contact zone dynamics in Wrangell-St. Elias

While ancestral areas tend to harbor older genotypes, areas with the greatest genetic diversity tend to be regions of secondary contact between previously disjunct groups [83]. The Wrangell-St. Elias region appears to be an important zone of secondary contact for alpine mammals, as genetic diversity in this area was higher than in any other area of our study. Wrangell-St. Elias NPP and the neighboring Kluane NPP in the Yukon Territory comprise the second-largest protected area in the world [84]. We know relatively little about the mammals of this region, with several new species records revealed by recent inventory work [85]. In-depth ecological and evolutionary studies of Wrangell-St. Elias mammals are needed to reveal more about the role glacial margins and nunataks play in preserving and structuring diversity of alpine organisms and the colonization of previously glaciated regions by these species. The high genetic divergence in Singing Voles and Brown Lemmings may be the result of limited introgression between divergent phylogroups [86]. This is supported by the fact that Eastern and Beringian clades have not been found in sympatry in either species, suggesting a role for competitive exclusion, residual allopatry of mitochondrial lineages, and/or gaps in sampling. For Collared Pikas and Hoary Marmots, lower intraspecific divergences and haplotype sympatry suggest that competition and adaptive divergence between haplogroups is unlikely in these two species. From an ecological, evolutionary, and climate change perspective, the Wrangell-St. Elias region has much to offer in terms of future studies of divergence and introgression in alpine organisms.

### “Interglacial refugia” or cryptic refugia?

The cold, arid, windswept “mammoth-steppe” of Beringia during the Pleistocene (Fig. 1D) is thought to have been similar to communities of plants and animals currently found in the alpine and boreal tundra of Alaska and northern Canada [50,87]. Pleistocene fossils suggest that species of plants and animals that are now confined to disjunct alpine habitats were once widespread at lower elevations [87]. Bayesian skyline plots for four of the five species in our study support a history of glacial expansion as opposed to population contraction (Fig. 4), thus terming the ancestral locations for the recovered phylogroups as “refugia” is not appropriate. If refugia are considered to be the region of maximum contraction in the geographical range of a species (*sensu* [19]), then neither fossil nor demographic data support the paradigm of Pleistocene glacial refugia in these arctic/alpine species.

The demographic consequences of range shifts from the Pleistocene to the present are influenced by the degree of geographic shift as well as population structure. Small habitat patches at the LGM, especially in easternmost Beringia (e.g., Fig. 1D), may have prevented a significant loss of diversity [20]. First, the Pleistocene presence of glacial margin (periglacial or “peripheral” [26]) and nunatak populations could have preserved diversity, especially if dispersal between patches was low. Second, the LGM was a relatively short time period [88] when compared to the expansion and contraction phases of glacial cycling. Suitable habitat for alpine-adapted animals was more extensive during and immediately following the LGM [89] and has subsequently retracted.

For species that have experienced glacial population expansion and interglacial population contraction, it might be more appropriate to consider their current areas of occupation “interglacial refugia” [19]. While this may seem like a minor distinction, there may be heuristic value in the recognition that recent climatic trends are stressing species whose distributions and abundances were already declining. Recent range reduction and population extirpation reported in species such as the Olympic Marmot (*Marmota olympus*) [90] and American Pika (*Ochotona princeps*) [91] may presage decline in other alpine and arctic-adapted species. Skyline plots (Fig. 4) suggest post-Pleistocene declines are already underway for Arctic Ground Squirrels, Singing Voles, Collared Pikas.

In contrast, there is evidence of demographic expansion without subsequent population decline in Brown Lemmings (Fig. 4). The moderate Beringian fossil record for Brown Lemmings [92–94] indicates their presence across the region during the Wisconsin glaciation. Post-Pleistocene changes in available habitat appear to have driven demographic growth without subsequent contraction, perhaps because this species prefers mesic habitat [95], which has expanded since the end of the Pleistocene [96]. However, it remains to be seen if this post-Pleistocene expansion will be (or is being) checked by recent warming.

### Effective population size and population cycles

Differences in the intraspecific divergence among the taxa in our study also highlight the role that fecundity, vagility, and population fluctuation play in preserving genetic diversity and increasing effective population size [97]. The two most intraspecifically divergent species (Brown Lemmings and Singing Voles) have higher effective population sizes and undergo higher-amplitude population fluctuations (Fig. 3; S1 Table). Although the low cycles during population fluctuation are expected to decrease effective population size in local populations, effective sizes are still high relative to the lower-density, lower-amplitude Hoary Marmots, Collared Pikas, and Arctic Ground Squirrels. The multigenerational effective size of cyclic populations (calculated as the harmonic mean size over time [98]) should be closer to the low-phase population size than the high-phase, but will still be higher than the effective population sizes of other species in our study. For Brown Lemmings and Singing Voles, presumed higher mutation rates and increased dispersal during the high-phase of population cycles likely also play a role in maintaining genetic diversity within each clade [97]. Population diversity is shifting in response to climate change (e.g., [5,72], over both short and long timeframes, with possible consequences for future evolutionary potential. As a result, life history traits that increase effective population size over time could play a large role in maintaining adaptive potential in the face of global warming. Understanding these interactions is crucial to predicting the genetic responses to climate change in the future, particularly for taxa already undergoing population and/or range declines.

## Conclusions

Despite differences in ecology and demography, broadly similar phylogeographic histories are supported for all species, suggesting that these, and likely other, alpine- and arctic-adapted taxa in the region have been shaped by the same historical phenomena. This includes strong support for contemporaneous population divergences across all five species and support for more recent demographic declines in three species. Such declines may be synergistically accelerating due to ongoing rapid climate change. Thus, the habitat affinities that promoted divergence in these species during past episodes of climate change may be leading to the erosion of genetic diversity as populations are confined to increasingly fragmented and isolated alpine habitats.

## Supporting Information

S1 File Supporting Figures A-E: Maximum-likelihood trees. (PDF)

S2 File Supporting Tables A-E: GenBank accession numbers. (PDF)

S1 Table Major ecological and life history differences among species. (PDF)
