# Supplementary material for: Comparative Phylogeography Highlights the Double-Edged Sword of Climate Change Faced by Arctic- and Alpine-Adapted Mammals
Source: PLoS One. 2015 Mar 3;10(3):e0118396. doi: 10.1371/journal.pone.0118396 (PMC4348485; doi:10.1371/journal.pone.0118396)

S3 Supporting Figures

Figure A - Brown lemming (*Lemmus trimucronatus*) maximum likelihood tree constructed using the TIM2+I+G model

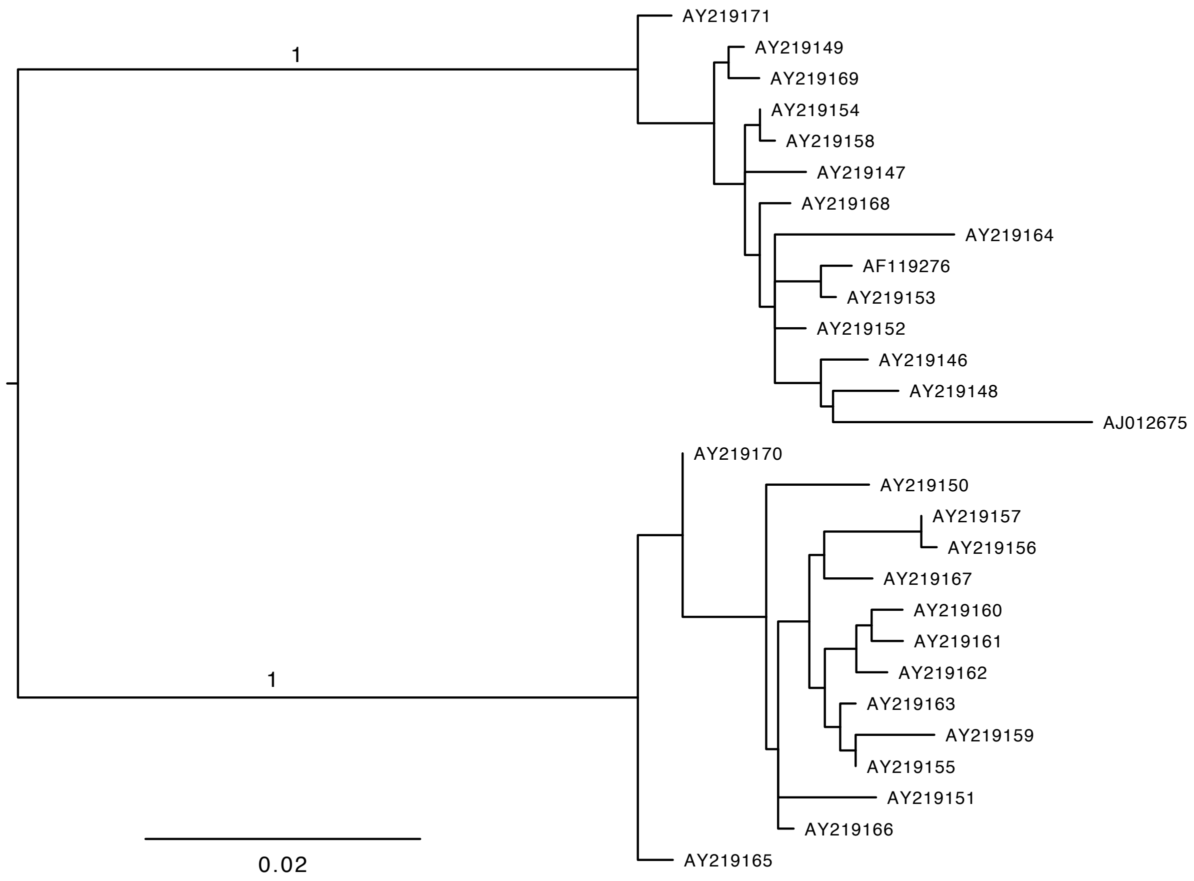

Figure B - Hoary marmot (*Marmota caligata*) maximum likelihood tree constructed using the TrN+G model

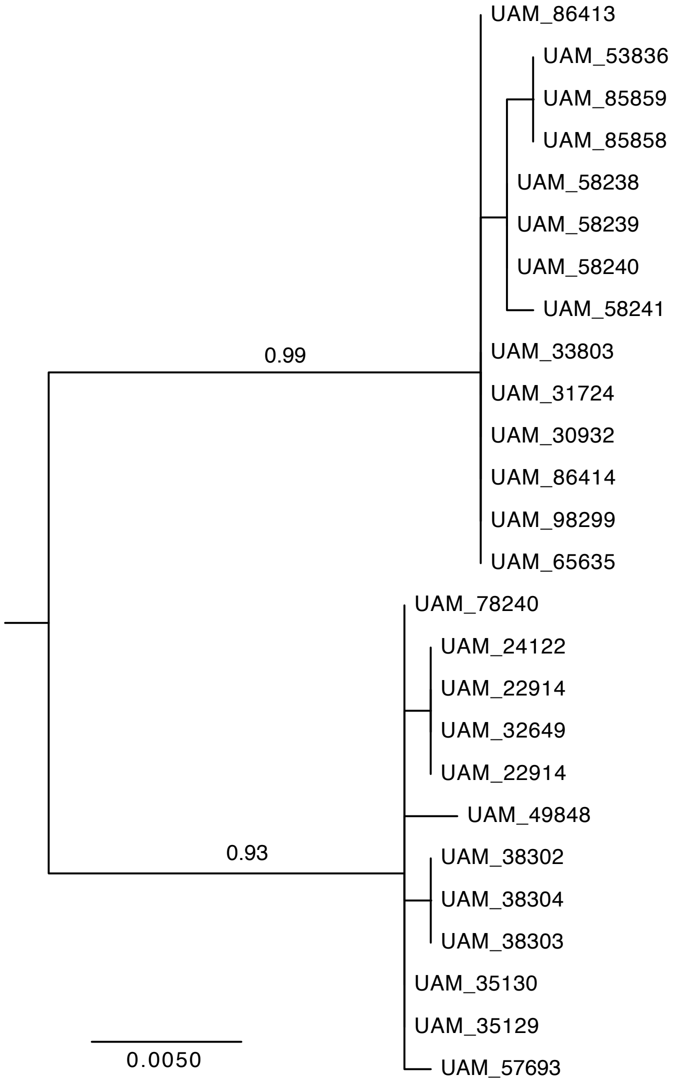

Figure C - Singing vole (*Microtus miurus*) maximum likelihood tree constructed using the TIM1+I model

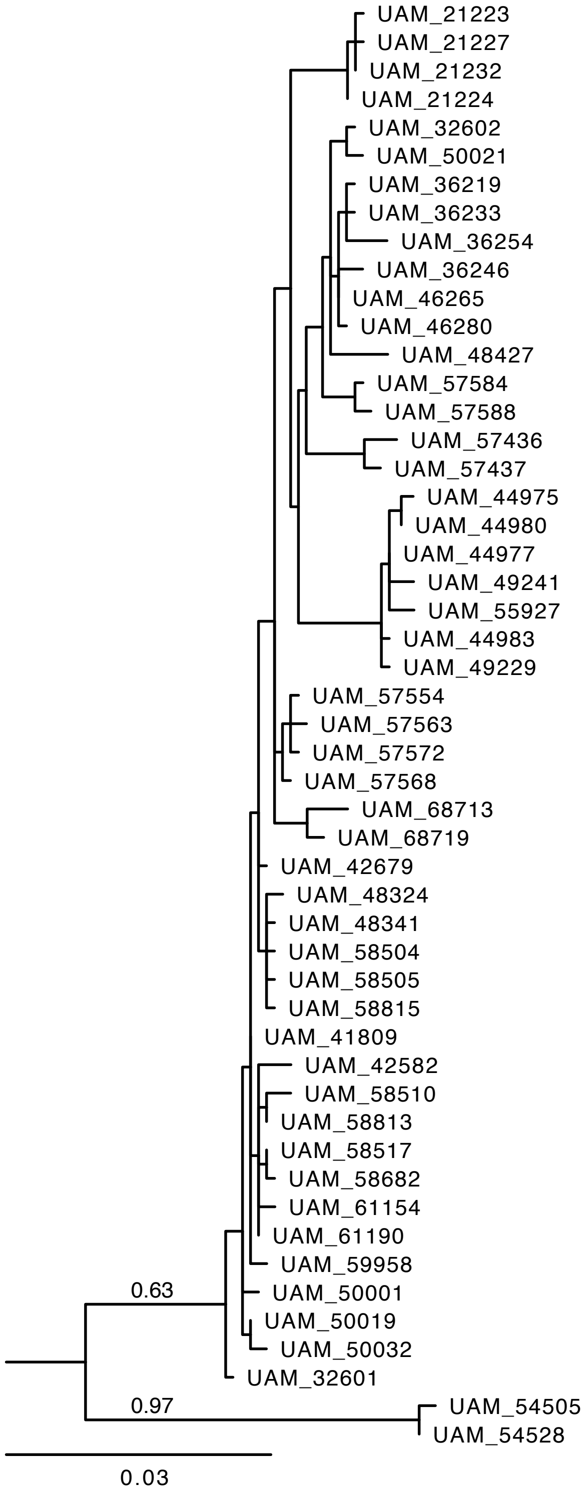

Figure D - Collared pika (*Ochotona collaris*) maximum likelihood tree constructed using the TIM3+I+G model

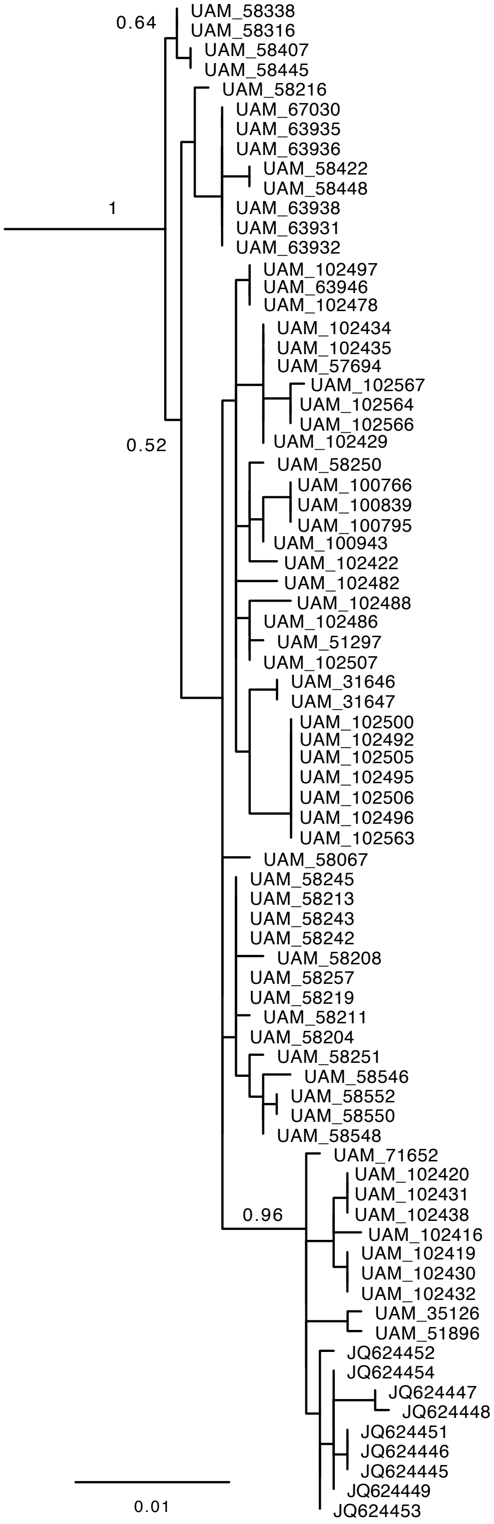

Figure E - Arctic ground squirrel (*Urocitellus parryii*) maximum likelihood tree constructed using the TIM1+I+G model

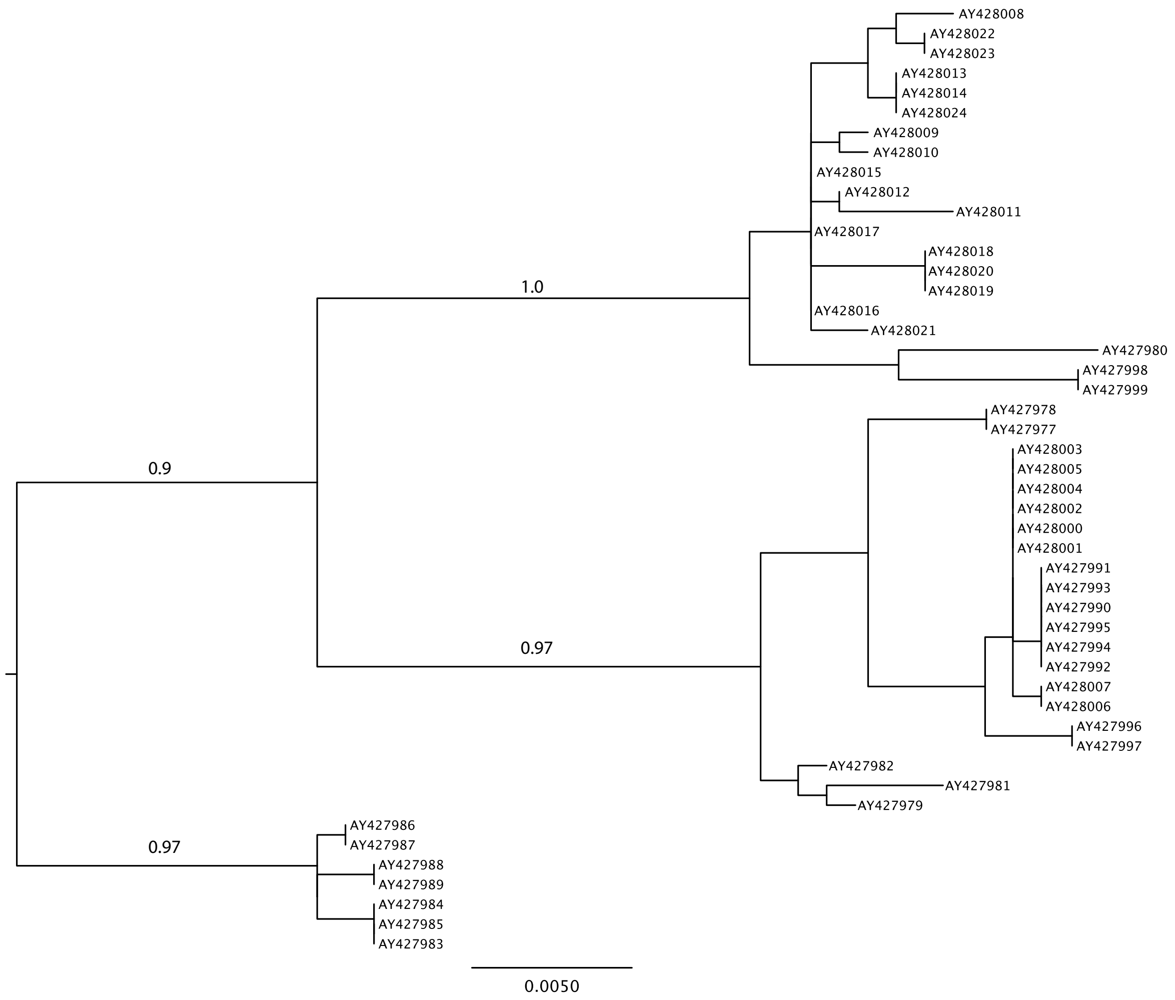

Supplement: S1 File — (PDF) [file pone.0118396.s001.pdf]
