# Supplementary material for: Comparative Phylogeography Highlights the Double-Edged Sword of Climate Change Faced by Arctic- and Alpine-Adapted Mammals
Source: PLoS One. 2015 Mar 3;10(3):e0118396. doi: 10.1371/journal.pone.0118396 (PMC4348485; doi:10.1371/journal.pone.0118396)
Supplement: S2 File — (PDF) [file pone.0118396.s002.pdf]

S1 Supporting Tables

Table A: GenBank accession number for Brown Lemming samples used in this study. Samples are sorted by species and haplogroup. *Lemmus sibericus* () was used as an outgroup. *Lemmus trimucronatus* (AJ012675) from East of Kolyma Delta was used as an outgroup.

**Brown Lemming** (*Lemmus trimucronatus* [13])			
West *n* = 14		East *n* = 14	
AJ012675	AY219154	AY219170	AY219160
AY219171	AY219147	AY219167	AY219159
AY219164	AY219153	AY219166	AY219157
AY219169	AF119276	AY219165	AY219156
AY219149	AY219148	AY219163	AY219155
AY219168	AY219146	AY219162	AY219151
AY219158	AY219152	AY219161	AY219150

Table B: GenBank accession number and specimen ID (UAM = University of Alaska Museum) for Hoary Marmot samples used in this study (new accessions sequenced by AMG using methods outlined in [14]). Samples are sorted by species and haplogroup. *Marmota flaviventris* (AF143926 and AF143927) and *M. olympus* (AF143935) were used as outgroups.

**Hoary Marmot** (*Marmota caligata* [15,16])	
West *n* = 14	East *n* = 12
KJ458208 - UAM 30932	AF143920 - UAM 22914
KJ458232 - UAM 31724	FJ438937 - UAM 35130
KJ458229 - UAM 33803	FJ438938 - UAM 38304
KJ458218 - UAM 53836	FJ438939 - UAM 49848
KJ458214 - UAM 58238	FJ438940 - UAM 57693
KJ458215 - UAM 58239	FJ438941 - UAM 78240
KJ458216 - UAM 58240	KJ458225 - UAM 22914
KJ458217 - UAM 58241	KJ458224 - UAM 24122
KJ458222 - UAM 65635	KJ458226 - UAM 32649
KJ458209 - UAM 85858	KJ458223 - UAM 35129
KJ458210 - UAM 85859	KJ458221 - UAM 38302
KJ458211 - UAM 86413	KJ458227 - UAM 38303
KJ458212 - UAM 86414	
KJ458213 - UAM 98299	

Table C: GenBank accession number and specimen ID (UAM = University of Alaska Museum) for Singing Vole samples used in this study. Samples are sorted by species and haplogroup. Starred (*) individuals excluded from the msBayes analyses but included in summary statistic calculations.

**Singing Vole** (*Microtus miurus* [17])	
West n = 35	
GU809077 - UAM 68097*	GU809132 - UAM 64503
GU809081 - UAM 68104*	GU809133 - UAM 64389*
GU809089 - UAM 51869	GU809137 - UAM 64406*
GU809092 - UAM 53080	GU809138 - UAM 64353
GU809094 - UAM 75332*	GU809139 - UAM 64357*
GU809096 - UAM 75345*	GU809143 - UAM 81971
GU809101 - UAM 75427	GU809142 - UAM 64375
GU809104 - UAM 76019	GU809152 - UAM 81979
GU809105 - UAM 76039*	GU809153 - UAM 79610
GU809107 - UAM 78042*	GU809155 - UAM 79805*
GU809109 - UAM 78051	GU809159 - UAM 78877
GU809111 - UAM 78108*	GU809160 - UAM 78878*
GU809113 - UAM 78041	GU809161 - UAM 78692
GU809115 - UAM 56150	GU809162 - UAM 98727
GU809117 - UAM 56154*	GU809170 - UAM 56352
GU809119 - UAM 56383*	GU809171 - UAM 98356
GU809121 - UAM 56354	East n = 2
GU809124 - UAM 85562	GU809129 - UAM 57749
GU809125 - UAM 85818*	GU809130 - UAM 57771

Table D: GenBank accession number and specimen ID (UAM = University of Alaska Museum; Hik = sample ID from the Hik lab at the University of Alberta Edmonton) for Collared Pika samples used in this study. Specimens subsampled from contemporary populations utilized in Lanier & Olson, 2013 [18], to ensure coverage of populations from east and west clades. Starred (*) individuals excluded from msBayes analyses but included in summary statistic calculations. *Ochotona princeps* (AF176579 and AF272989) was used as an outgroup.

**Collared Pika** (*Ochotona collaris* [18])	
West n = 58	
EU549743 - UAM 57694	JQ624487 - UAM 102478*
EU549739 - UAM 58445	JQ624489 - UAM 102482
EU549740 - UAM 63936*	JQ624491 - UAM 102486*
JQ624524 - UAM 31646	JQ624493 - UAM 102488*
JQ624525 - UAM 31647*	JQ624495 - UAM 102492*
JQ624529 - UAM 56067	JQ624498 - UAM 102495*
JQ624535 - UAM 58204*	JQ624499 - UAM 102496*
JQ624538 - UAM 58208*	JQ624500 - UAM 102497*
JQ624539 - UAM 58211	JQ624502 - UAM 102500*
JQ624541 - UAM 58213*	JQ624507 - UAM 102505*
JQ624542 - UAM 58216	JQ624508 - UAM 102506
JQ624543 - UAM 58219*	JQ624509 - UAM 102507*
JQ624544 - UAM 58242	JQ624510 - UAM 102554*
JQ624545 - UAM 58243	JQ624517 - UAM 102564
JQ624547 - UAM 58245*	JQ624519 - UAM 102566*
JQ624550 - UAM 58250*	JQ624520 - UAM 102567
JQ624551 - UAM 58251*	KP411018 - UAM 51297*
JQ624552 - UAM 58257*	KP411019 - UAM 102562*
JQ624554 - UAM 58316*	KP411020 - UAM 102563*
JQ624555 - UAM 58338*	East n = 19
JQ624558 - UAM 58407*	EU549742 - UAM 35126
JQ624559 - UAM 58422*	EU549737 - UAM 71652
JQ624562 - UAM 58448*	JQ624527 - UAM 51896
JQ624563 - UAM 58546	JQ624474 - UAM 102416
JQ624565 - UAM 58548*	JQ624475 - UAM 102419
JQ624567 - UAM 58550*	JQ624476 - UAM 102420
JQ624569 - UAM 58552	JQ624481 - UAM 102430
JQ624571 - UAM 63931	JQ624482 - UAM 102431
JQ624572 - UAM 63932*	JQ624483 - UAM 102432
JQ624573 - UAM 63938	JQ624486 - UAM 102438
JQ624575 - UAM 67030*	JQ624451 - Hik 441
JQ624460 - UAM 100776	JQ624452 - Hik 446
JQ624461 - UAM 100795	JQ624453 - Hik 492
JQ624465 - UAM 100839*	JQ624454 - Hik 499
JQ624472 - UAM 100943*	JQ624445 - Hik 1355
JQ624477 - UAM 102422*	JQ624446 - Hik 1385
JQ624480 - UAM 102429*	JQ624447 - Hik 1555
JQ624484 - UAM 102434*	JQ624448 - Hik 1628
JQ624485 - UAM 102435	JQ624449 - Hik 1649

Table E: GenBank accession number and, where applicable, specimen ID (UAM = University of Alaska Museum) in parentheses for Artic Ground Squirrel samples used in this study. Samples are sorted by species and haplogroup. *Urocitellus beecheyi* (AF157919 and AF157918) was used as an outgroup.

**Arctic Ground Squirrel** (*Urocitellus parryii* [19])		
West *n* = 21	East *n* = 20	North *n* = 7
AY427977 - UAM 50603	AY427980 - UAM 34208	AY427989 - UAM 49909
AY427978 - UAM 50604	AY428008 - UAM 51300	AY427988 - UAM 49898
AY427979 - UAM 31887	AY428009 - UAM 35165	AY427987 - UAM 97406
AY427981 - UAM 51312	AY428010 - UAM 35167	AY427986 - UAM 49900
AY427982 - UAM 72215	AY428011 - UAM 63968	AY427985 - UAM 53905
AY427990 - UAM 24347	AY428012 - UAM 64087	AY427984 - UAM 53904
AY427991 - UAM 24127	AY428013 - UAM 64090	AY427983 - UAM 53903
AY427992 - UAM 24126	AY428014 - UAM 64088	
AY427993 - UAM 24121	AY428015 - UAM 31903	
AY427994 - UAM 24123	AY428016 - UAM 31902	
AY427995 - UAM 24125	AY428017 - UAM 32935	
AY427996 - UAM 43112	AY428018 - UAM 22027	
AY427997 - UAM 54611	AY428019 - UAM 24124	
AY428000 - UAM 35040	AY428020 - UAM 51389	
AY428001 - UAM 43095	AY428021 - UAM 35163	
AY428002 - UAM 43096	AY428022	
AY428003 - UAM 50841	AY428023	
AY428004 - UAM 43097	AY428024	
AY428005 - UAM 50845	AY427998 - UAM 32776	
AY428006 - UAM 43098	AY427999 - UAM 32777	
AY428007 - UAM 43099		

References

1. MacDonald SO, Cook JA (2009) Recent Mammals of Alaska. Fairbanks, Alaska: University of Alaska Press. 387 p.
2. Gillis EA, Hik DS, Boonstra R, Karels TJ, Krebs CJ (2005) Being high is better: Effects of elevation and habitat on arctic ground squirrel demography. Oikos 108: 231-240.
3. Zgurski JM, Hik DS (2012) Polygynandry and even-sexed dispersal in a population of collared pikas, *Ochotona collaris*. Anim Behav 83: 1075-1082.
4. Barash DP (1973) Marmots: social behavior and ecology. Stanford: Stanford University Press. 368 p.
5. Kyle CJ, Karels TJ, Davis CS, Mebs S, Clark B, Strobeck C, Hik DS (2007) Social structure and facultative mating systems of hoary marmots *(Marmota caligata*). Mol Ecol 16: 1245-1256.
6. Batzli GO, Henttonen H (1993) Home range and social organization of the singing vole (*Microtus miurus*). J Mammal 74: 868-878.
7. Stenseth NC, Ims RA (1993) The biology of lemmings. London: Academic Press. 704 p.
8. McLean IG. (1983) Paternal behaviour and killing of young in Arctic ground squirrels. Anim Behav 31: 32-44.
9. Franken RJ, Hik DS (2004) Interannual variation in timing of parturition and growth of collared pikas (*Ochotona collaris*) in southwest Yukon. Int Comp Biol 44: 186-193.
10. Batzli GO, Henttonen H (1990) Demography and resource use by microtine rodents near Toolik Lake, Alaska, USA. Arctic Alpine Res 22: 51-64.
11. Franken RJ, Hik DS. (2004) Influence of habitat quality, patch size and connectivity on colonization and extinction dynamics of collared pikas *Ochotona collaris*. J Animal Ecol 73: 889-896.
12. Erhich D, Jorde PE (2005) High genetic variability despite high-amplitude population cycles in lemmings. J Mammal 86: 380-385.
13. Fedorov VB, Goropashnaya AV, Jaarola M, Cook JA (2003) Phylogeography of lemmings (*Lemmus*): No evidence for postglacial colonization of Arctic from the Beringian refugium. Mol Ecol 12: 725-731.
14. Gunderson AM, Lanier HC, Olson LE (2012) Limited phylogeographic structure and genetic variation in Alaska's arctic and alpine endemic, the Alaska marmot. J Mammal 93: 66-75.
15. Steppan SJ, Akhverdyan MR, Lyapunove EA, Fraser DG, Vorontsov NN, Hoffmann RS, Braun MJ (1999) Molecular phylogeny of the marmots (Rodentia: Sciuridae): tests of evolutionary and biogeographic hypotheses. Syst Biol 48: 715-734.
16. Gunderson AM, Jacobsen BK, Olson LE (2009) Revised distribution of the Alaska Marmot, *Marmota broweri*, and confirmation of parapatry with Hoary Marmots. J Mammal 90: 859-869.
17. Weksler M, Lanier HC, Olson LE (2010) Eastern Beringian biogeography: historical and spatial genetic structure of singing voles in Alaska. J Biogeog 37: 1414-1431.

18. Lanier HC, Olson LE (2013) Deep barriers, shallow divergences: reduced phylogeographical structure in the collared pika (Mammalia: Lagomorpha: *Ochotona collaris*). J. Biogeog 40: 466-478.
19. Eddingsaas AA, Jacobsen BK, Lessa EP, Cook JA (2004) Evolutionary history of the arctic ground squirrel (*Spermophilus parryii*) in Nearctic Beringia. J Mammal 85: 601-610.
