# Supplementary material for: Comparative Phylogeography Highlights the Double-Edged Sword of Climate Change Faced by Arctic- and Alpine-Adapted Mammals
Source: PLoS One. 2015 Mar 3;10(3):e0118396. doi: 10.1371/journal.pone.0118396 (PMC4348485; doi:10.1371/journal.pone.0118396)
Supplement: S1 Table — (PDF) [file pone.0118396.s003.pdf]

S1 Table – Ecological and demographic differences

Species	Body mass[a]	Habitat[1]	Mating system	Reproductive output	Age at maturity	Population structure
Collared Pika	114-135 g	alpine talus	polygynandry[3]	1 litter per year[9]; 2 to 4 young per litter[9]	1 year[11]	relatively stable metapopulation[11]
Hoary Marmot	3.7-5.6 kg	alpine meadow	monogamy, polygamy[4,5]	0.5 to 1 litter per year[4,5]; 2 to 5 young per litter[4,5]	3+ years[4]	metapopulation or source-sink
Singing Vole	15-35 g	alpine or arctic tundra	promiscuous[6]	> 1 litter per year[10]; 6 to 8 young per litter[a]	<1 year[10]	variable colonies (10x amplitude) [6]
Brown Lemming	26-42 g	alpine, arctic, & maritime tundra	promiscuous[7]	> 1 litter per year; active in winter[7]; 4 to 6 young per litter[a]	<1 year[7]	50 – 125x high amplitude cycles[10,12]
Arctic Ground Squirrel	250-621 g	alpine & arctic tundra; boreal forest[2]	promiscuous[8]	1 litter per year[8]; 6 to 8 young per litter[2]	1 year[2]	source-sink dynamics[2]

[a.]From 1st to 3rd quartiles based on University of Alaska Museum specimen records
